# Supplementary figures and images for: Multi-cell ECM compaction is predictable via superposition of nonlinear cell dynamics linearized in augmented state space
Source: PLoS Comput Biol. 2019 Sep 20;15(9):e1006798. doi: 10.1371/journal.pcbi.1006798 (PMC6774565; doi:10.1371/journal.pcbi.1006798)

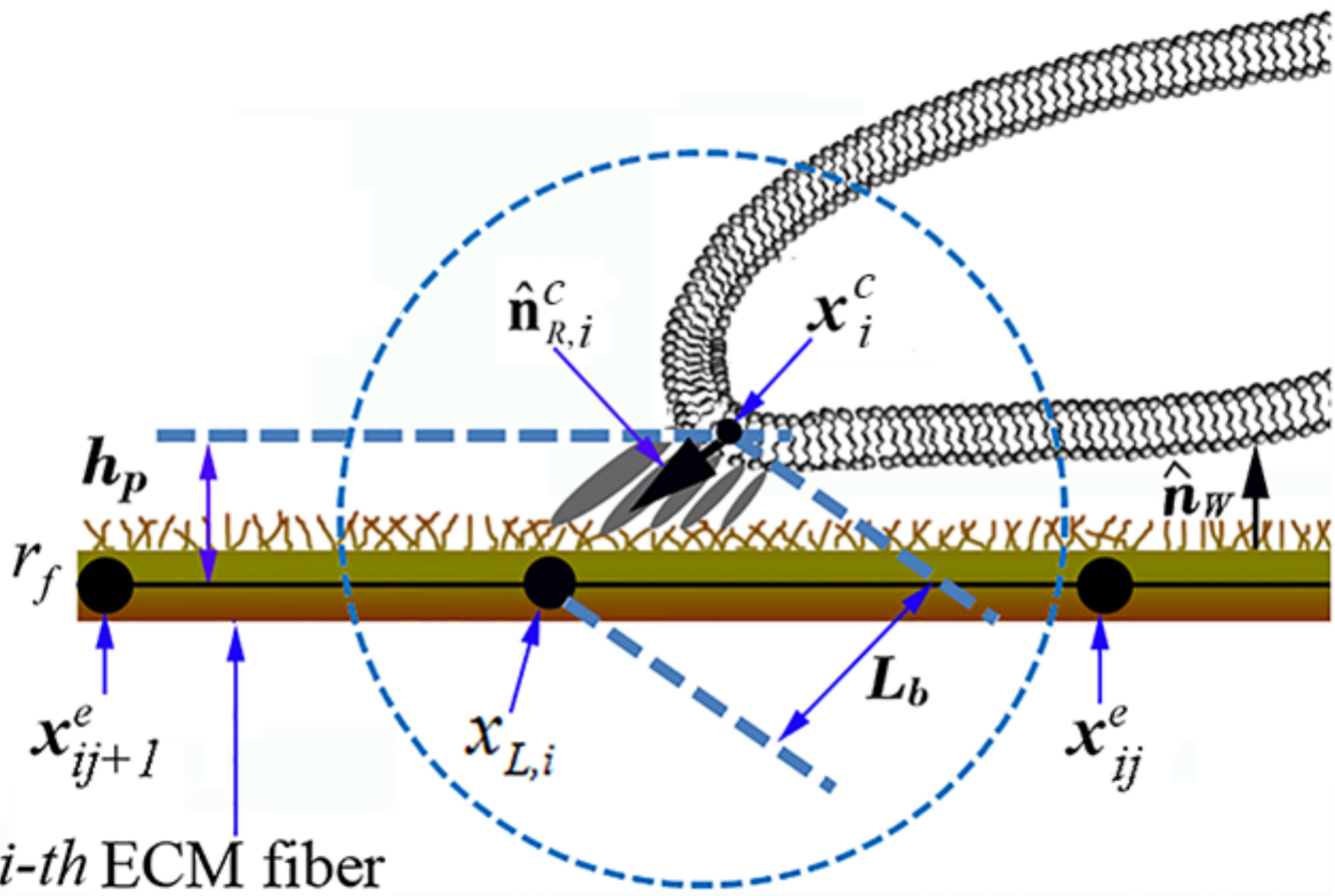

Supplement: S1 Fig — Schematic showing integrin molecules on the cellular membrane interacting with an extracellular matrix fiber, and illustrating a stochastic ligand-receptor bonding process at the focal adhesion site. (PDF) [file pcbi.1006798.s001.pdf]

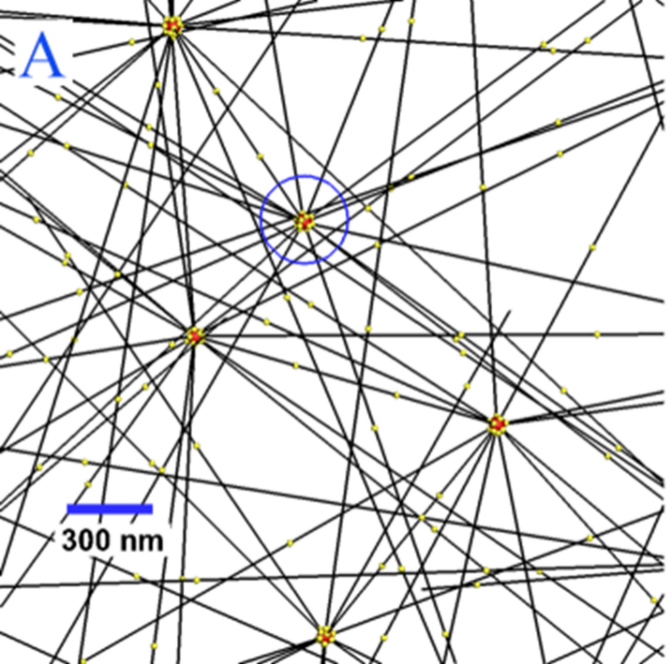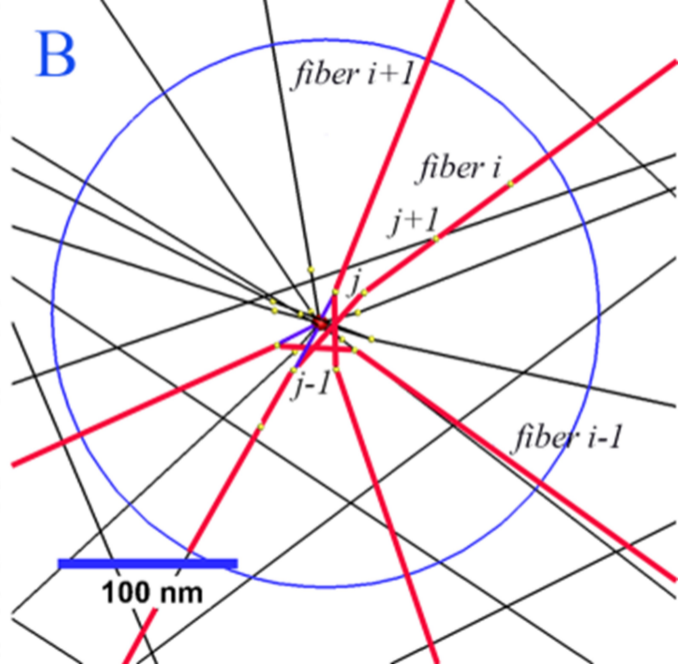

Supplement: S2 Fig — A: Segmented ECM fibers were generated between crosslink nodes. Yellow spheres indicate segmented ECM fiber nodes. B: A magnified view in blue circle mark in A showing examples of three fibers’ connectivity with a crosslink node. Blue lines indicated crosslinks between an ECM fiber node and a crosslink node. (PDF) [file pcbi.1006798.s002.pdf]
