# Supplementary material for: Multi-cell ECM compaction is predictable via superposition of nonlinear cell dynamics linearized in augmented state space
Source: PLoS Comput Biol. 2019 Sep 20;15(9):e1006798. doi: 10.1371/journal.pcbi.1006798 (PMC6774565; doi:10.1371/journal.pcbi.1006798)
Supplement: S1 Text — (PDF) [file pcbi.1006798.s007.pdf]

Supplementary Information for

Multi-Cell ECM compaction is predictable via superposition of nonlinear cell dynamics linearized in augmented state space

Michaëlle N Mayalu, Min-Cheol Kim and H. Harry Asada

Michaëlle N Mayalu and H. Harry Asada
E-mail: mmayalu@caltech.edu and asada@mit.edu

This PDF file includes:

Supplementary text
References for SI reference citations

Other supplementary materials for this manuscript include the following:

Figures S1-S2
Videos S1-S4

Supplementary Text

Appendix A. Nonlinear Dynamics of Cell-ECM Interaction for Computational Model

Our nonlinear computational model is composed of two modules: 1) intracellular mechanics including focal adhesion dynamics, actin motor activity, and mechanics of cellular and nuclear membranes, and 2) dynamics of ECM fiber network. The detailed equations that govern each of these dynamical processes are summarized in the following sections, and the list of simulation parameters are also summarized in supplementary Table S1.

1) Intracellular mechanics. The intracellular mechanics is a key mechanism involved in cell interactions within a 3D ECM fiber network. The essential equations in the model consist of: a) equations describing focal adhesion dynamics based on forward and backward kinetics of ligand-receptor bonds, b) and equation for lamellipodium protrusion by actin polymerization, and c) an equation for cortical stress and elastic energy force. These governing equations are extensions of the prior works(1, 2) with improvements for streamlining computation and modeling validity specifically for the current work.

a). Focal Adhesion Dynamics

As shown in Fig. S1, the Focal adhesion (FA) force acts between the $i-th$ integrin node on the cellular membrane and points on the ECM fibers where the extension of the unit vector normal to the cellular membrane interacts with the nearest point of ECM fibers. Focal adhesion (FA) force, $\mathbf{F}^c_{FA,i}$, at the $i-th$ node of the outer cell membrane is expressed as:

$$\mathbf{F}^c_{FA,i} = n_{b,i}\kappa_{LR}\left(L_b - \lambda\right)\hat{\mathbf{n}}^c_{R,i} \quad [1]$$

where $n_{b,i}$is the number of integrin-collagen bonds,$\kappa_{LR}$is the spring constant of a single ligand-receptor bond (1 pN/nm) (3) , $L_b$is the average stretched length of the ligand-receptor bonds, λ is an unstressed length of bonds (30nm) (4) , and $\hat{\mathbf{n}}^c_{R,i}$is a unit vector at the local surface of the $i-th$ node of the outer cell membrane toward the bonding site at the ECM fiber (Fig. S1). For brevity, superscript k, indicating the cell's number, is omitted in the following derivation. We use Bell's model(5) to incorporate force-dependent reaction rates of the number of bonds ($n_{b,i}$), which is expressed with the following ordinary differential equation:

$$\frac{dn_{b,i}}{dt} = k_{on}\left(n_{tot} - n_{b,i}\right) - k_{off}n_{b,i} \quad [2]$$

where $n_{tot}$is total available number of integrin molecules at the $i-th$ node of cellular membrane, $k_{on}$is the kinetic associate rate for binding integrin molecules and ECM fiber, and it is expressed as (6, 7):

$$k_{on} = k^0_{on}\frac{l_{bind}}{Z_0}\exp\left[-\frac{\kappa_{LR}(L_b - \lambda)^2}{2k_bT}\right] \quad [3]$$

where k^0_{on} is the zero forward reaction rate ($1\ molecule^{-1}\ s^{-1}$, $l_{bind}$is a binding radius (30 nm) to check whether the $i-th$ node of cellular membrane and the node on the fiber are sufficiently close, and $k_bT$is the unit of thermal energy. The parameter $Z_0$is the partition function for an integrin molecule confined in a harmonic potential between $-\lambda$and $L_b - \lambda$, and is expressed as

$$Z_0 = \sqrt{\frac{\pi k_bT}{2\kappa_{LR}}}\left(erf\left[(L_b - \lambda)\sqrt{\frac{\kappa_{LR}}{2k_bT}}\right] + erf\left[\lambda\sqrt{\frac{\kappa_{LR}}{2k_bT}}\right]\right) \quad [4]$$

The parameterk_{off} in Eq. 2 is the kinetic dissociation rate. It is known as Bell's equation for the slip bond, given by (5):

$$k_{off} = k^0_{off}\exp\left[\frac{\kappa_{LR}\left(L_b - \lambda\right)x_b}{k_bT}\right] \quad [5]$$

where $k^0_{off}$is the zero kinetic dissociation rate in the absent of the force, $x_b$is the distance between the minimum binding potential and the transition state barrier, and ${k_bT}/{x_b}$ represents an intrinsic force $200pN$. From Fig. S1 the root location of receptor - ligand bonds ($\boldsymbol{x}_{L,i}$) is given by

$$\boldsymbol{x}_{L,i} = \boldsymbol{x}^c_i + L_b\hat{\mathbf{n}}_{R,i} = \boldsymbol{x}^c_i - \frac{h_p\,\hat{\mathbf{n}}_{R,i}}{\hat{\mathbf{n}}_w\cdot\hat{\mathbf{n}}_{R,i}}. \quad [6]$$

where $\hat{\mathbf{n}}_w$is the unit vector orthogonal to the ECM fiber, and $h_p$is the gap between the $i-th$ node of cellular membrane and the ECM fiber. This expression is valid only when $\hat{\mathbf{n}}_w\cdot\hat{\mathbf{n}}_{R,i} < 0$and the gap $h_p$is less than a critical height (h_c) of 300 nm (<10λ):$h_p < h_c$. The latter condition is to restrict the formation of receptor-ligand bonds within the upper limith_c.

b). Lamellipodium Force

The lamellipodium force at the $i-th$ node of the outer cell membrane, $\mathbf{F}^c_{L,i}$, is generated at the leading edge of migratory cells. It is deemed the actual motors pushing the cortical cytoskeleton forward during the process of cell migration (8) . Normally, cells experience a small protrusive pressure that results from osmotic pressure or actin branches stimulated by activated arp2/3. Here we assume that the magnitude of the lamellipodium force is constant at 300 pN, and exists at only leading edges of the cell.

c). Cortical Force and Elastic Energy Force

The cortical force and elastic energy force, collectively represented with $\mathbf{F}^{c,k}_{Cort-Elas}$, comprise both elastic and damping forces generated at the membrane attached to actin cortex and further connected to the nucleus through actin stress fibers. We model this with a three-layer mesh structures: outer cell membrane layer, inner transduce layer, and nucleus layer. The computational model is threefold:

(i) The cell membrane layer exhibits elastic energy force, which is modelled using elastic energy stored in the mesh structure;

(ii) The Kelvin-Voigt model is applied to the outer membrane and inner transduce layers to represent the viscoelastic behavior between the two layers; and

(iii) Between the inner transduce layer and the nucleus layers actin stress fibers are formed, which exhibit elastic force and contractile force.

i) Elastic energy forces at the membrane layer

The elastic force at the $i-th$ node of the outer cell membrane, $\mathbf{F}^{c}_{E,i}$, is obtained by using the virtual work theory in structural mechanics. To this end, the total elastic energy stored in the cell membrane is obtained. Two types of total elastic energy are considered. One is the total elastic energy associated with distance changes between nodes (9, 10):

$$H^c_L = \frac{\kappa^c_L}{2}\sum_{i=1}^{line}\left(L^c_i - L^{c0}_i\right)^2 \qquad [7]$$

where L^c_i is the length of the $i-th$ line of the cell membrane mesh, which is updated at every time-step, $L^{c0}_i$is its unstressed length, κ^c_L is effective stiffness of the line elements connecting the cell membrane nodes (5.0×10^{-5} N/m) (11, 12). Similarly, the total elastic energy associated with area changes in the membrane nodal mesh is given by

$$H^c_A = \frac{\kappa^c_A}{2}\sum_{i=1}^{element}\left(\frac{A^c_i - A^{c0}_i}{A^{c0}_i}\right)^2 A^{c0}_i \qquad [8]$$

where$A^c_i$is the $i-th$ mesh area of the cell membrane and $A^{c0}_i$is its unstressed values, and κ^c_A is an effective stiffness constant of area elements of the cell membrane ($1.0\times10^{-4} N/m^2$) (10) Then, $\mathbf{F}^c_{E,i}$can be obtained by differentiating the total of the two types of elastic energy,

$$\mathbf{F}^c_{E,i} = -\frac{\partial H^c_L}{\partial \boldsymbol{x}^c_i} - \frac{\partial H^c_A}{\partial \boldsymbol{x}^c_i} \qquad [9]$$

ii). The double-layer Kelvin-Voigt model

Including the elastic force at the outer membrane layer, we can write the combined cortical and elastic force as:

$$\mathbf{F}^c_{Cort-Elas,i} = \mathbf{F}^c_{E,i} + \mathbf{F}^c_{T,i} + D_{Cort}\left(\frac{d\boldsymbol{x}^t_i}{dt} - \frac{d\boldsymbol{x}^c_i}{dt}\right) \qquad [10]$$

where $\mathbf{F}^c_{T,i}$is a transduce force representing the elastic force of actin cortex in the Kelvin-Voigt model at the $i-th$ node of the outer cell membrane. It is given by

$$\mathbf{F}^c_{T,i} = -\kappa_{cort}\left(L_{T,i} - L^0_{T,i}\right)\frac{\partial L_{T,i}}{\partial \boldsymbol{x}^c_i} \qquad [11]$$

where $\kappa_{cort}$is an effective spring constant of line element of the actin cortex (8.0×10^{-3} N/m), $L_{T,i}$ is the length of the $i-th$ line in the actin cortex, which is updated at every time-step, and $L^0_{T,i}$is its unstressed length (500 nm). The damping term in the Kelvin-Voigt model is proportional to the difference in velocity between the two layers, $d\boldsymbol{x}^t_i/dt - d\boldsymbol{x}^c_i/dt$, where $\boldsymbol{x}^t_i$is the coordinates of the $i-th$ node on the inner transduce layer. The dynamic equation of the inner transduce membrane can be expressed as

$$D_t\frac{d\boldsymbol{x}^t_i}{dt} + D_{cort}\left(\frac{d\boldsymbol{x}^t_i}{dt} - \frac{d\boldsymbol{x}^c_i}{dt}\right) = \mathbf{F}^t_{E,i} + \mathbf{F}^t_{SF,i} + \mathbf{F}^t_{T,i}, \quad i = 1,\cdots,N_t \qquad [12]$$

where $D_t$is a coefficient of dissipation energy for the inner transduce membrane (0.001 Ns/m), $\mathbf{F}^t_{T,i}$ is a transduce force at the $i-th$ node of the inner transduce membrane, which balances with $\mathbf{F}^c_{T,i} = -\mathbf{F}^t_{T,i}$, and $\mathbf{F}^t_{E,i}$ is an elastic force at the $i-th$ node of the inner transduce membrane. Similarly to the above analysis, two kinds of total elastic energy stored in the inner transduce membrane are considered. One is the total elastic energy associated with distance changes between the nodes:

$$H^t_L = \frac{\kappa^t_L}{2}\sum_{i=1}^{line}\left(L^t_i - L^{t0}_i\right)^2 \qquad [13]$$

where κ_L^t is effective stiffness of the line elements of the inner membrane (5.0×10^{-5} N/m), L_i^t is the length of the $i-th$ line of the inner membrane mesh updated at every time-step, and $L_i^{t0}$is its unstressed length. Similarly, the total elastic energy associated with area changes is given by

$$H_A^t = \frac{\kappa_A^t}{2} \sum_{i=1}^{element} \left(\frac{A_i^t - A_i^{t0}}{A_i^{t0}} \right)^2 A_i^{t0} \qquad [14]$$

where$A_i^t$is the $i-th$ mesh area of the inner membrane and $A_i^{t0}$is its unstressed values, κ_A^t is effective stiffness of area elements of the inner membrane ($1.0 \times 10^{-4} N/m^2$). Then, $\mathbf{F}_{E,i}^t$can be obtained by differentiating the two kinds of total energy,

$$\mathbf{F}_{E,i}^t = -\frac{\partial H_L^t}{\partial \boldsymbol{x}_i^t} - \frac{\partial H_A^t}{\partial \boldsymbol{x}_i^t} \qquad [15]$$

iii). Actin Stress Fiber Contraction

In the dynamic equation of the inner transduce layer, $\mathbf{F}_{SF,i}^t$is an actin stress fiber (SF) force at the $i-th$ node of the inner transduce membrane. The actin SF is a bundle of actin microfilaments assembled by actin-myosin II interactions. In the model, the $i-th$ node of the inner transduce membrane is connected to the $j-th$ node of nuclear membrane by a SF. Its connection to the $j-th$ node of nuclear membrane is determined by the nearest distance from the $i-th$ node of the inner membrane to the nucleus. The stiffness of a SF is a variable. According to literature, the stiffness increases with a contractile agonist (histamine) and decreases with a relaxing agonist (isoproterenol)(13). These characteristics must be reflected to the formulation of the SF stiffness:

$$\kappa_{SF} = \frac{E_{SF} A_{SF}}{L_{SF,i}^1} \qquad [16]$$

where $E_{SF}$is Young's modulus of SFs (230 kPa) directly measured from isolated smooth muscle cells(14), A_{SF} is the average cross-sectional area of SFs (250 nm in radius (15), and $L_{SF,i}^1$is the length of a single compartment of the $i-th$ SF. Similarly, the elastic energy stored in the $i-th$ SF is given by

$$\begin{aligned} E_{SF,i} &= \sum_{j=1}^{N_{SF}} \left[\tfrac{\kappa_{SF}}{2} \left(\tfrac{d_{SF,i}}{N_{SF}} - L_{SF,j}^1 \right)^2 \right] \\ &= \tfrac{\kappa_{SF}}{2} \left(\tfrac{d_{SF,i}}{N_{SF}} - L_{SF,1}^1 \right)^2 N_{SF} = \tfrac{\kappa_{SF}}{2N_{SF}} \left(d_{SF,i} - N_{SF} L_{SF,1}^1 \right)^2 \end{aligned} \qquad [17]$$

where N_{SF} is the number of contractile compartments in the $i-th$ SF, and $d_{SF,i}$represents the distance between $i-th$ node of inner membrane and $j-th$ node of nuclear membrane for a SF connected to the nucleus. It should be noted that $d_{SF,i}$physically means the length of SFs under tension and $L_{SF,1}^1$ represents the length of a single unstressed bundle of SFs. Using the virtual work theory, forces due to actin SFs' motor activity at the $i-th$ node of inner membrane and the $j-th$ node of nuclear membrane is given by

$$\mathbf{F}_{SF,i}^t = -\frac{\partial E_{SF,i}}{\partial \boldsymbol{x}_i^t} = -\frac{\kappa_{SF}}{N_{SF}} \left(d_{SF,i} - N_{SF} L_{SF,i}^1 \right) \frac{\partial d_{SF,i}}{\partial \boldsymbol{x}_i^t} \qquad [18]$$

It is considered that actin motor activity starts when the other end of a SF is connected to the nucleus, and ends when integrin nodes are broken from FAs. The sliding rate of myosin II is known to fluctuate (i.e. is non-uniform) unlike myosin I which slides with a uniform rate. Furthermore, the sliding rate of myosin II is adjusted by sensing the transmitted focal adhesion force from the ECM (16). To incorporate these characteristics into the model, force-velocity relation of muscle myosin II, first proposed by A.V. Hill (17), is adopted as the following equation:

$$v_m = v_{m0} \frac{F_{m0} - F_{FA}}{F_{m0} + c_m F_{FA}} \qquad [19]$$

where $v_{m0}$is the sliding rate of myosin in the absence of load (10 nm/s), F_{m0} is the isometric force of myosin, or stall force, and c_m is a parameter in the force-velocity relationship for myosin. Initially, the length of sarcomere unit is 800 nm ($L_{SF,i}^1 = 800$ nm at t = 0 s), which contracts until 60 % of the initial length has contracted. As the contraction takes place at both sides of each sarcomere unit, the minimum time required for 60 % contraction is calculated as 16 s with v_{m0}. Actin motor activity is terminated when integrin nodes are broken from FAs.

Lastly, the dynamic equation at the $i-th$ node of the nuclear membrane can be expressed as

$$D_n \frac{d\boldsymbol{x}_i^n}{dt} = \mathbf{F}_{E,i}^n + \mathbf{F}_{SF,i}^n, \quad i = 1, \cdots, N_n \qquad [20]$$

where $D_n$is a coefficient of dissipation energy for the nuclear membrane (0.001 Ns/m), $\boldsymbol{x}_i^n$is a position vector at the $i-th$ node in the membrane of nucleus, and $\mathbf{F}_{E,i}^n$is an elastic force at the $i-th$ node of the nuclear membrane. Similarly, two

kinds of total elastic energy stored in the nuclear membrane are considered. One is the total elastic energy associated with distance changes between the nodes (9, 10):

$$H_L^n = \frac{\kappa_L^n}{2}\sum_{i=1}^{line}\left(L_i^n - L_i^{n0}\right)^2 \qquad [21]$$

where κ_L^n is effective stiffness of the line elements of the nuclear membrane (5.0×10^{-3} N/m) (18) (50), L_i^n is the length of the $i-th$ line of the nuclear membrane mesh, which is updated at every time-step, and takes a constant value$L_i^{n0}$when it is unstressed. Similarly, the total elastic energy associated with area changes is given by

$$H_A^n = \frac{\kappa_A^n}{2}\sum_{i=1}^{element}\left(\frac{A_i^n - A_i^{n0}}{A_i^{n0}}\right)^2 A_i^{n0} \qquad [22]$$

where$A_i^n$is the $i-th$ mesh area of the nuclear membrane and $A_i^{n0}$is its relaxed values. κ_A^n is an effective stiffness constant of area elements of the nuclear membrane ($1.0 \times 10^{-4} N/m^2$). Then, $\mathbf{F}_{E,i}^n$can be obtained by differentiating the two kinds of total energy,

$$\mathbf{F}_{E,i}^n = -\frac{\partial H_L^n}{\partial \boldsymbol{x}_i^n} - \frac{\partial H_A^n}{\partial \boldsymbol{x}_i^n}. \qquad [23]$$

Integrating the above models, we can simulate the intracellular dynamics in great detail. Combined with the dynamics of ECM fiber network, as detailed below, detailed nonlinear simulations can produce a data set of $\boldsymbol{x}_i^c, \boldsymbol{x}_i^e, \mathbf{F}_{Cort-Elas,i}^c$,$\mathbf{F}_{FA,i}^c$,$\mathbf{F}_{L,i}^c$, $\mathbf{F}_{Elas,i}^e$, as defined in the main text. In the DF Linearization, regression models are formed based on the simulation data, instead of performing the detailed nonlinear simulation. The detailed dynamics of the inner transduce membrane and nucleus layers as well as the stress fiber dynamics are imbedded in the regression models in Eqs. (10) and (11). This significantly reduces the computational load.

2) Dynamics of ECM fiber network. We assume the ECM fiber network to be composed of viscoelastic ECM fibers and crosslinks, which make strong bonds between adjacent fibers(19). The elastic energy stored in the ECM fiber network can be expressed in terms of stretching and bending properties of the constituent fibers. The stretching modulus of a fiber is given by $\kappa_{f,s}^e \left(= E_f^e A_f\right)$, where $E_f^e$and $A_f \left(= \pi r_f^2\right)$ are Young's modulus (1 Mpa) and the cross-sectional area of a single fiber, respectively. The bending modulus of a fiber is given by$\kappa_{f,b}^e \left(= E_f I_f\right)$, where $I_f \left(= \pi r_f^4/4\right)$ (20). The stretching elastic energy of the $j-th$ segment of the $i-th$ fiber is given as a function of the difference between the stressed $\left(L_j^{e,i}\right)$ and unstressed $\left(L_{j0}^{e,i}\right)$ lengths, and the bending elastic energy as the one of stressed $\left(\theta_j^{e,i}\right)$ and unstressed $\left(\theta_{j0}^{e,i}\right)$ angles at the $j-th$ node between two segments in the $i-th$ fiber (Fig. S2). The total elastic energy in the $i-th$ ECM fiber in the network can be expressed as:

$$\mathrm{H}_f^{e,i} = \frac{\kappa_{f,s}^e}{2}\sum_{j=1}^{N_i^e}\frac{\left(L_j^{e,i} - L_{j0}^{e,i}\right)^2}{L_{j0}^{e,i}} + \frac{\kappa_{f,b}^e}{2}\sum_{j=1}^{N_i^e}\frac{\left(\theta_j^{e,i} - \theta_{j0}^e\right)^2}{L_{j0}^{e,i}}. \qquad [24]$$

Here, it should be noted that the elastic energy at the $j-th$ node in the $i-th$ fiber is summed only for coaxial neighboring nodes. Similarly, the elastic force at the $j-th$ node in the $i-th$ fiber,$bfF_{E,i\,j}^e$, can be derived by using the virtual work principle:

$$\mathbf{F}_{E,j}^{e,i} = -\frac{\partial \mathrm{H}_f^{e,i}}{\partial \boldsymbol{x}_j^{e,i}} = -\kappa_{f,s}^e\sum_{k=j}^{j+1}\frac{\left(L_k^{e,i} - L_{k0}^{e,i}\right)}{L_k^{e0,i}}\frac{\partial L_k^{e,i}}{\partial \boldsymbol{x}_j^{e,i}} - \kappa_{f,b}^e\sum_{k=j-1}^{j+1}\frac{\left(\theta_k^{e,i} - \theta_{k0}^{e,i}\right)}{L_k^{e,i}}\frac{\partial \theta_k^{e,i}}{\partial \boldsymbol{x}_j^{e,i}} \qquad [25]$$

where $\theta_k^{e,i} = \cos^{-1}\left(\hat{t}_k^i \cdot \hat{t}_{k+1}^i\right)$,$\hat{t}_k^i$and $\hat{t}_{k+1}^i$are tangential unit vectors at the k and k+1-st nodes in the $i-th$ fiber, respectively, and $\frac{\partial \theta_k^{e,i}}{\partial \boldsymbol{x}_j^{e,i}} = \frac{-1}{\sqrt{1-\left(\hat{t}_k^i \cdot \hat{t}_{k+1}^i\right)^2}}\left(\frac{\partial \hat{t}_k^i}{\partial \boldsymbol{x}_j^{e,i}} \cdot \hat{t}_{k+1}^i + \hat{t}_k^i \cdot \frac{\partial \hat{t}_{k+1}^i}{\partial \boldsymbol{x}j^{e,i}}\right)$. To solve the dynamics of ECM fiber network, a dynamic equation at the $i-th$ ECM fiber node can be expressed as

$$D_e\frac{d\boldsymbol{x}_i^e}{dt} = \mathbf{F}_{FA,i}^e + \mathbf{F}_{E,i}^e\,, \quad i = 1, \cdots, N_e. \qquad [26]$$

where D_e is a coefficient of dissipation energy for the ECM fiber, and $\mathbf{F}_{FA,i}^e$is a FA force at the $i-th$ ECM fiber node. Note that dynamics of ECM fibers is coupled with intracellular mechanics through the relationship: $\mathbf{F}_{FA,i}^e + \mathbf{F}_{FA,i}^c = 0$.

Appendix B. Least Squares Estimation for Identification of the Parameter Matrices$\mathbf{A}, \mathbf{B}, \mathbf{C}, \mathbf{G}$ involved in the Latent Space State Equations

The Dual Faceted Linearization method discussed in this work is used to represent the nonlinear system in augmented space with two sets of linear differential equations:

$$\left.\begin{array}{l}\frac{d\boldsymbol{x}^{c,k}}{dt} = \mathbf{W}_{CE}^c\mathbf{F}_{Cort-Elas}^{c,k} + \mathbf{W}_{FA}^c\mathbf{F}_{FA}^{c,k} + \mathbf{L}_c\mathbf{u}^k, \quad k = 1, \cdots, n_{cell} \\ \frac{d\boldsymbol{x}^e}{dt} = \mathbf{W}_{Elas}^e\mathbf{F}_{Elas}^e + \mathbf{W}_{FA}^e\mathbf{F}_{FA}^e\end{array}\right\} set\ 1 \qquad [27]$$

$$\left.\begin{array}{l} \frac{d\mathbf{F}^{c,k}_{Cort-Elas}}{dt} \simeq \mathbf{Q}^c_x \boldsymbol{x}^{c,k} + \mathbf{Q}^c_{F_{CE}} \mathbf{F}^{c,k}_{Cort-Elas} + \mathbf{Q}_u \mathbf{u}^k, \quad k = 1, \cdots, n_{cell} \\ \frac{d\mathbf{F}^{c,k}_{FA}}{dt} \simeq \mathbf{H}^c_x \boldsymbol{x}^{c,k} + \mathbf{H}^e_x \boldsymbol{x}^e + \mathbf{H}^c_{F_{FA}} \mathbf{F}^{c,k}_{FA} + \mathbf{H}_u \mathbf{u}^k \\ \frac{d\mathbf{F}^e_{Elas}}{dt} \simeq \mathbf{R}^e_x \boldsymbol{x}^e + \mathbf{R}^e_{F_{Elas}} \mathbf{F}^e_{Elas} \end{array}\right\} set\ 2 \qquad [28]$$

The first set of differential equations Eq. 27 are the original state equations, which are apparently linear in terms of the auxiliary variables ($\mathbf{F}^{c,k}_{Cort-Elas}, \mathbf{F}^{c,k}_{FA}, \mathbf{F}^e_{Elas}$) and input$\mathbf{u}^k$. Since the ECM focal adhesion forces ($\mathbf{F}^e_{FA}$) can be represented as a linear compilation of cell membrane focal adhesion forces ($\mathbf{F}^{c,k}_{FA}$), they are excluded from the set of auxiliary variables. Here,$\boldsymbol{x}^{c,k} \in \Re^{3N_c \times 1}$ is a vector containing the 3-D coordinates $\left(\boldsymbol{x}^{c,k}_i\ i = 1, \ldots, N_c\right)$ of all the cell membrane nodes and $\boldsymbol{x}^e_i \in \Re^{3N_e \times 1}$ is a vector containing the 3-D coordinates ($\boldsymbol{x}^e_i\ i = 1, \ldots, N_e$) of all the ECM nodes. $\mathbf{F}^{c,k}_{Cort-Elas} \in \Re^{3N_c \times 1}$ is a vector that comprises cortical tension and elastic energy forces ($\mathbf{F}^{c,k}_{Cort-Elas,i}$) for all the cell nodes. $\mathbf{F}^{c,k}_{FA} \in \Re^{3N_c \times 1}$is a vector of focal adhesion forces ($\mathbf{F}^{c,k}_{FA,i}$) at all the cell nodes. Variable $\mathbf{u}^k \in \Re^{3N_c \times 1}$ is an input vector containing all the lamellipodium forces ($\mathbf{F}^{c,k}_{L,i}$). $\mathbf{W}^c_{CE}, \mathbf{W}^c_{FA}$,$\mathbf{L_C}$,$\mathbf{W}^e_{Elas}$, $\mathbf{W}^e_{FA}$are constant matrices of consistent dimensions. The second set of differential equations (Eq. 28) represent the transition of auxiliary state variables estimated through linear regressions. Here, ($\mathbf{R}^*_* \in \Re^{3N_e \times 3N_e}$,$\mathbf{Q}^*_* \in \Re^{3N_c \times 3N_c}$,$\mathbf{H}^*_* \in \Re^{3N_c \times 3N_c}$) are high-dimensional parameter matrices. As discussed in the main text, we transform the augmented linearized system to the one in the latent variable space spanned by eigenvectors ($\mathbf{V}^c = \begin{pmatrix} \mathbf{V}^{c\,\mathbf{T}}_x & \mathbf{V}^c_{F_{CE}}{}^{\mathbf{T}} & \mathbf{V}^c_{F_{FA}}{}^{\mathbf{T}} \end{pmatrix}^{\mathbf{T}} \in \Re^{m_c \times m_c}$and $\mathbf{V}^e = \begin{pmatrix} \mathbf{V}^{e\,\mathbf{T}}_x & \mathbf{V}^e_{F_{Elas}}{}^{\mathbf{T}} \end{pmatrix}^{\mathbf{T}} \in \Re^{m_e \times m_e}$) of the covariance matrices, as detailed in Method S3 below.

$$\frac{d\mathbf{z}^{c,k}}{dt} = \mathbf{A}\,\mathbf{z}^{c,k} + \mathbf{B}\,\mathbf{u}^k + \mathbf{C}\mathbf{z}^e, \quad k = 1, \cdots, n_{cell} \qquad [29]$$

$$\frac{d\mathbf{z}^e}{dt} = \mathbf{G}\,\mathbf{z}^e + \sum_{k=1}^{n_{cell}} \mathbf{D}^k \mathbf{z}^{c,k} \qquad [30]$$

where:

$$\begin{array}{l} \mathbf{A} = \mathbf{V}^{c\,\mathbf{T}}_x (\mathbf{W}^c_{CE} \mathbf{V}^c_{F_{CE}} + \mathbf{W}^c_{FA} \mathbf{V}^c_{F_{FA}}) + \mathbf{V}^c_{F_{CE}}{}^{\mathbf{T}} (\mathbf{Q}^c_x \mathbf{V}^c_x + \mathbf{Q}^c_{F_{CE}} \mathbf{V}^c_{F_{CE}}) + \mathbf{V}^c_{F_{FA}}{}^{\mathbf{T}} (\mathbf{H}^c_x \mathbf{V}^c_x + \mathbf{H}^c_{F_{FA}} \mathbf{V}^c_{F_{FA}}) \\ \mathbf{B} = \mathbf{V}^{c\,\mathbf{T}}_x \mathbf{L}_c + \mathbf{V}^c_{F_{CE}}{}^{\mathbf{T}} \mathbf{Q}_u + \mathbf{V}^c_{F_{FA}}{}^{\mathbf{T}} \mathbf{H}_u \\ \mathbf{C} = \mathbf{V}^{\mathbf{T}}_{F_{FA}}{}^c \mathbf{H}^e_x \mathbf{V}^e_x \\ \mathbf{G} = \mathbf{V}^{e\,\mathbf{T}}_x \mathbf{W}^e_{Elas} \mathbf{V}^e_{F_{Elas}} + \mathbf{V}^e_{F_{Elas}}{}^{\mathbf{T}} (\mathbf{R}^e_x \mathbf{V}^e_x + \mathbf{R}^e_{F_{Elas}} \mathbf{V}^e_{F_{Elas}}) \\ \mathbf{D}^k = \mathbf{V}^{e\,\mathbf{T}}_x \mathbf{W}^e_{FA} \mathbf{P}^k_{map} \mathbf{V}^c_{F_{FA}} \end{array} \qquad [31]$$

Here, $\mathbf{P}^k_{map} \in \Re^{3N_e \times 3N_c}$is a parameter matrix (consisting of either 0 or -1 elements) which maps the membrane focal adhesion forces of the k-th cell ($\mathbf{F}^{c,k}_{FA}$) to the corresponding ECM focal adhesion forces ($\mathbf{F}^e_{FA}$) as discussed in the main text. Since the system is represented in a lower dimensional space, the high dimensional regression coefficient matrices ($\mathbf{R}^*_*, \mathbf{Q}^*_*, \mathbf{H}^*_*$) are not computed explicitly. Instead, the lower dimension coefficient matrices $\mathbf{A}, \mathbf{B}, \mathbf{C}, \mathbf{G}$ are computed directly from numerical simulation data transformed into the latent variable space. We define transformed data set $\mathrm{Z}_{Tr} = \left\{\left(\mathbf{z}^{e,n}(t), \mathbf{z}^{c,k,n}(t), \mathbf{u}^{k,n}(t), d\mathbf{z}^{e,n}/dt, d\mathbf{z}^{c,k,n}/dt\right) | k = 1, \cdots, K,\ n = 1, \cdots, N,\ t = 1, \cdots, T\right\}$. Here superscripts k, n signify the k-th cell ($K = 1$ or $K = 2$) within the n-th simulation. We combine parameter matrices from equation Eq. 29 into $\mathrm{M} \triangleq \begin{bmatrix} \mathbf{A} & \mathbf{B} & \mathbf{C} \end{bmatrix} \in \Re^{m_c \times (m_c + N_c + m_e)}$and variables into $\xi^{k,n}(t) = \begin{pmatrix} \mathbf{z}^{c,k,n}(t)^{\mathbf{T}} & \mathbf{u}^{k,n}(t)^{\mathbf{T}} & \mathbf{z}^{e,n}(t)^{\mathbf{T}} \end{pmatrix}^{\mathbf{T}} \in \Re^{(m_c + N_c + m_e) \times 1}$. The parameter matrix M can be optimized so that the mean squared error of predicting $d\mathbf{z}^{c,k,n}/dt$ may be minimized:

$$\mathrm{M}^0 = \arg\min_{\mathrm{M}} \frac{1}{K \cdot N \cdot T} \sum_{k=1}^{K} \sum_{n=1}^{N} \sum_{t=1}^{T} \left\| \left. \frac{d\mathbf{z}^{c,k,n}}{dt} \right|_t - \mathrm{M}\xi^{k,n}(t) \right\|^2 \qquad [32]$$

Using the standard least squared estimation and assuming that the sample data sufficiently spans the dimension of vector $\xi^{k,n}(t)$, we can obtain:

$$\mathrm{M}^0 = \left(\sum_{k=1}^{K} \sum_{n=1}^{N} \sum_{t=1}^{T} \left. \frac{d\mathbf{z}^{c,k,n}}{dt} \right|_t \xi^{k,n}(t)^{\mathbf{T}} \right) \left(\sum_{k=1}^{K} \sum_{n=1}^{N} \sum_{t=1}^{T} \xi^{k,n}(t)\, \xi^{k,n}(t)^{\mathbf{T}} \right)^{-1} \qquad [33]$$

Similarly least squares estimate matrix $\mathbf{G}$ is given by:

$$\mathbf{G}^0 = \left(\sum_{n=1}^{N} \sum_{t=1}^{T} \delta^n(t)\, \mathbf{z}^{e,n}(t)^{\mathbf{T}} \right) \left(\sum_{n=1}^{N} \sum_{t=1}^{T} \mathbf{z}^{e,n}(t)\, \mathbf{z}^{e,n}(t)^{\mathbf{T}} \right)^{-1} \qquad [34]$$

where $\delta^n(t) = d\mathbf{z}^{c,k,n}/dt\big|_t - \sum_{k=1}^{K} \mathbf{D}^k \mathbf{z}^{c,k,n}$ and $\mathbf{D}^k$'s are known matrices as defined in Eq.31.

Appendix C. Implementing Polarity Model and Lamellipodial Force Generation

The polarity direction of a cell is important for determining the orientation of the leading edge that rotates dynamically in response to changes in local ECM stiffness. Implementing the polarity model that leads to the generation of lamellipodial forces on the leading edge requires two functional relations. One is to relate the direction of maximum ECM stiffness $\mathbf{d}^{e,k}_{Max-Stiff}$ to global stresses within the ECM, which depend on latent variable state vector $\mathbf{z}^e$. The other is to relate the direction of polarity vector,$\mathbf{d}^k_{Pol}$, to the lamellipodium forces of each membrane node.

For the former functional relation, the local stiffness of ECM fiber network changes depending on the global stress generated over the ECM. The latent variable state vector $\mathbf{z}^e$ pertains to this ECM property and, thereby, allows us to predict the direction of maximum stiffness. For the latent variable superposition model, we assume that the maximum stiffness direction can be determined by:

$$\mathbf{d}^{e,k}_{Max-Stiff} = K_{Stiff} \begin{pmatrix} \mathbf{z}^e \\ \boldsymbol{x}^k_{center} \end{pmatrix} \quad [35]$$

where $\boldsymbol{x}^k_{center} \in \Re^{3\times1}$ is the center of mass of the k-th cell, which is determined as the mean of all the node coordinates of the cell, and $\mathbf{K}_{Stiff} : \Re^{(m_E+3)\times1} \mapsto \Re^{3\times1}$ maps ECM latent variables and the cell's center location to the direction of the maximum stiffness. Details on the calculation of direction of maximum stiffness based on the nonlinear full-scale computational model are given in reference (2). The optimized coefficient matrix $\mathbf{K}_{Stiff}$ is estimated from numerical simulation data of the full-scale nonlinear equations. Using least squares:

$$\mathbf{K}^0_{Stiff} = \left(\sum_{k=1}^{K}\sum_{n=1}^{N}\sum_{t=1}^{T} \mathbf{d}^{e,k,n}_{Max-Stiff}(t)\,\chi^{k,n}(t)^{\mathbf{T}} \right) \left(\sum_{k=1}^{K}\sum_{n=1}^{N}\sum_{t=1}^{T} \chi^{k,n}(t)\,\chi^{k,n}(t)^{\mathbf{T}} \right)^{-1} \quad [36]$$

where $\chi^{k,n}(t) = \begin{pmatrix} \mathbf{z}^{e\mathbf{T}} & \boldsymbol{x}^{k\ \mathbf{T}}_{center} \end{pmatrix}^{\mathbf{T}}$and superscripts k and n represent the cell number and simulation iteration of the variable sample data as discussed previously.

In the latter functional relation, consider a right circular cone of apex angle $2\alpha^k_L$ shown in Fig. 3B in the main text. The centerline of the cone is aligned with the unit vector of polarity direction, $\mathbf{d}^k_{Pol}$. The cell's membrane nodes $\boldsymbol{x}^{c,k}_i$within this cone, where $0 \le \alpha^k_L < \pi/2$, are deemed the leading edge region of the cell, producing nonzero lamellipodial forces.

$$\mathbf{F}^{c,k}_{L,i} \begin{cases} \neq 0, & \mathbf{d}^{k\ T}_{Pol} \Delta\boldsymbol{x}^{c,k}_i \ge \left|\Delta\boldsymbol{x}^{c,k}_i\right| \cos\alpha^k_L \\ = 0, & \mathbf{d}^{k\ T}_{Pol} \Delta\boldsymbol{x}^{c,k}_i < \left|\Delta\boldsymbol{x}^{c,k}_i\right| \cos\alpha^k_L \end{cases} \quad [37]$$

where $\Delta\boldsymbol{x}^{c,k}_i = \boldsymbol{x}^{c,k}_i - \boldsymbol{x}^k_{center}$ is the position vector from the center point of the k-th cell to the i-th node of the cell's membrane.

References

1. Kim MC, Whisler J, Silberberg YR, Kamm RD, Asada HH. Cell Invasion Dynamics into a Three Dimensional Extracellular Matrix Fibre Network. PLOS Computational Biology. 2015;11(10):e1004535. doi:10.1371/journal.pcbi.1004535.
2. Kim MC, Silberberg YR, Abeyaratne R, Kamm RD, Asada HH. Computational modeling of three-dimensional ECM-rigidity sensing to guide directed cell migration. Proceedings of the National Academy of Sciences. 2018; p. 201717230.
3. Dembo M. On peeling an adherent cell from a surface. Lectures on Mathematics in the Life Sciences, Some Mathematical Problems in Biology. 1994;26:51–77.
4. Kanchanawong P, Shtengel G, Pasapera AM, Ramko EB, Davidson MW, Hess HF, et al. Nanoscale architecture of integrin-based cell adhesions. Nature. 2010;468(7323):580.
5. Bell GI. Models for the specific adhesion of cells to cells. Science. 1978;200(4342):618–627.
6. Qian J, Wang J, Lin Y, Gao H. Lifetime and strength of periodic bond clusters between elastic media under inclined loading. Biophysical Journal. 2009;97(9):2438–2445.
7. Erdmann T, Schwarz US. Bistability of cell-matrix adhesions resulting from nonlinear receptor-ligand dynamics. Biophysical journal. 2006;91(6):L60–L62.
8. Jeon J, Alexander NR, Weaver AM, Cummings PT. Protrusion of a Virtual Model Lamellipodium by Actin Polymerization: A Coarse-grained Langevin Dynamics Model. Journal of Statistical Physics. 2008;133(1):79–100. doi:10.1007/s10955-008-9600-5.
9. Tsubota Ki, Wada S, Yamaguchi T. Particle method for computer simulation of red blood cell motion in blood flow. Computer methods and programs in biomedicine. 2006;83(2):139–146.
10. Tsubota Ki, Wada S. Elastic force of red blood cell membrane during tank-treading motion: Consideration of the membrane's natural state. International Journal of Mechanical Sciences. 2010;52(2):356–364.
11. Drury JL, Dembo M. Aspiration of human neutrophils: effects of shear thinning and cortical dissipation. Biophysical Journal. 2001;81(6):3166–3177.
12. Honarmandi P, Lee H, Lang MJ, Kamm RD. A microfluidic system with optical laser tweezers to study mechanotransduction and focal adhesion recruitment. Lab on a chip. 2011;11(4):684–694.
13. Wang N, Tolic-Nørrelykke IM, Chen J, Mijailovich SM, Butler JP, Fredberg JJ, et al. Cell prestress. I. Stiffness and prestress are closely associated in adherent contractile cells. American Journal of Physiology-Cell Physiology. 2002;282(3):C606–C616.
14. Deguchi S, Ohashi T, Sato M. Tensile properties of single stress fibers isolated from cultured vascular smooth muscle cells. Journal of biomechanics. 2006;39(14):2603–2610.
15. Lu L, Oswald SJ, Ngu H, Yin FCP. Mechanical properties of actin stress fibers in living cells. Biophysical journal. 2008;95(12):6060–6071.
16. Walcott S, Sun SX. A mechanical model of actin stress fiber formation and substrate elasticity sensing in adherent cells. Proceedings of the National Academy of Sciences. 2010;107(17):7757–7762.
17. Hill AV. The heat of shortening and the dynamic constants of muscle. In: Proc. R. Soc. Lond. B. vol. 126. The Royal Society; 1938. p. 136–195.
18. Zeng Y, Yip AK, Teo SK, Chiam KH. A three-dimensional random network model of the cytoskeleton and its role in mechanotransduction and nucleus deformation. Biomechanics and modeling in mechanobiology. 2012;11(1-2):49–59.
19. Stein AM, Vader DA, Weitz DA, Sander LM. The micromechanics of three-dimensional collagen-I gels. Complexity. 2011;16(4):22–28.
20. Yang L, Van der Werf KO, Fitié CF, Bennink ML, Dijkstra PJ, Feijen J. Mechanical properties of native and cross-linked type I collagen fibrils. Biophysical journal. 2008;94(6):2204–2211.
