# Supplementary material for: Multi-cell ECM compaction is predictable via superposition of nonlinear cell dynamics linearized in augmented state space
Source: PLoS Comput Biol. 2019 Sep 20;15(9):e1006798. doi: 10.1371/journal.pcbi.1006798 (PMC6774565; doi:10.1371/journal.pcbi.1006798)
Supplement: S1 Table — (PDF) [file pcbi.1006798.s008.pdf]

Table S1. List of simulation parameters.

Parameter	Definition	Value	Sources
A	Area [$\mu\ m^2$]		
A_f	Averaged cross-sectional area of a single fiber [$\mu\ m^2$]	$(1.32) \times 10^{-3}$	
A_i^c	Area of the $i-th$ surface of the cell membrane [$\mu\ m^2$]		
A_i^t	Area of the $i-th$ surface of the transduce layer [$\mu\ m^2$]		
A_i^n	Area of the $i-th$ surface of the nuclear membrane [$\mu\ m^2$]		
D_c	Friction coefficients associated with the energy dissipation at the integrin node [Nsm^{-1}]	0.001	C
D_{cort}	Drag coefficients associated with viscoelastic behaviors in actin cortex	0.006	C
D_e	Friction coefficients associated with the energy dissipation at the ECM fiber node [Nsm^{-1}]	0.001	C
D_t	Friction coefficients associated with the energy dissipation at the transduce node [Nsm^{-1}]	0.001	C
$d_{SF,i}$	Distance between the $i-th$ node of transduce layer and the $j-th$ node of nuclear membrane		
F	Force [N]		
E_f^e	Young's modulus value of single fiber [Mpa]	1	C
E_{SF}	Young's modulus value of SFs [kPa]	230	(14)
H	Energy	300	C
h_c	Critical height [nm]	300	C
h_p	Height from the surface of ECN fiber to the $i-th$ node of cellular membrane [nm]		
L	Length		
L_i^c	Length of the $i-th$ line on the surface of the cell membrane [μ m]	0.001	C
L_i^t	Length of the $i-th$ line on the surface of the transduce layer [μm]		
L_i^n	Length of the $i-th$ line on the surface of the nuclear membrane[μm]	0.001	C
L_b	Stretched length of bonds between receptors and ligands		
$L_{SF,i}^1$	Length of the $i-th$ single unit of SFs at the present time [nm]		
$L_{SF,i}^0$	Length of the $i-th$ single unit of SFs at the previous time [nm]		
$L_{i\,j}^e$	Stressed length of the $j-th$ segment of the $i-th$ fiber [µm]		
$L_{i\,j}^{e0}$	Unstressed length of the $j-th$ segment of the $i-th$ fiber [µm]		
N_e	Number of nodes at ECM fiber networks	30k $\sim$ 234k	
N_c	Number of nodes at cellular membrane	98	
N_t	Number of nodes at transduce layer	98	
N_n	Number of nodes at nuclear membrane	98	
N_{SF}	Number of contractile compartments in the $i-th$ SF		
N_i^e	Number of nodes at the $i-th$ fiber		
$n_{b,i}$	Number of bonds at between receptors on the $i-th$ node of cellular membrane and ligands		
n_{tot}	Total number of integrin molecules at each node of cellular membrane	100	C
$\hat{\mathbf{n}}_{R,i}^c$	A unit vector at the local surface of the $i-th$ node of cellular membrane		
$\hat{\mathbf{n}}_w$	A unit normal vector orthogonal to the ECM fiber		
κ_A^c	Effective spring constant of area elements of the cell membrane [N/m]	1.0×10^{-4}	(8)
κ_L^c	Effective spring constant of line elements of the cell membrane [N/m]	5.0×10^{-5}	(10,11)
κ_A^t	Effective spring constant of area elements of the transduce layer [N/m]	1.0×10^{-4}	C
κ_L^t	Effective spring constant of line elements of the transduce layer [N/m]	5.0×10^{-5}	C
κ_A^n	Effective spring constant of area elements of the nuclear membrane [N/m]	1.0×10^{-4}	(10,11)
κ_L^n	Effective spring constant of line elements of the nuclear membrane [N/m]	5.0×10^{-3}	(18)
κ_{cort}	Effective spring constant of line element of the actin cortex [N/m]	8.0×10^{-3}	C
$\kappa_{f,s}^e$	The stretching modulus of a fiber [nN]	0.615 $\sim$ 1.32	C
$\kappa_{f,b}^e$	The bending modulus of a fiber [pN μm^2]	$(3.02 \sim 12.81) \times 10^{-3}$	C
κ_{SF}	Effective stiffness of the $i-th$ single unit of SFs [N/m]		
κ_{LR}	Spring constant of a single ligand-receptor bond [pN/nm]	1.0	(3)
k_{on}	Kinetic association rate [s-1]		
k_{on}^0	Kinetic association rate at an unstressed state [$molecule^{-1}s^{-1}$]	1.0	C
k_{off}	Kinetic dissociation rate [s^{-1}]		
k_{off}^0	Kinetic dissociation rate at an unstressed state [s^{-1}]	1.0	C
$\theta_{i\,j}^e$	Stressed angle at the $j-th$ node between two segments in the $i-th$ fiber		
$\theta_{i\,j}^{e0}$	Unstressed angle at the $j-th$ node between two segments in the $i-th$ fiber		

$\hat{t}_{i,k}$	Tangential unit vector at the k-th segment in the $i-th$ fiber		
t	Time [s]		
ν_m	Sliding rate of non-muscle myosin II on the actin filaments [nm/s]		
$\boldsymbol{x}$	Location vector [μ m]		
$\boldsymbol{x}_{L,i}$	Root of ligand-rector bonds on the local surface of the fiber		
$\boldsymbol{x}^e_{ij}$	The $j-th$ location vector along to the $i-th$ fiber [μ m]		
λ	Equilibrium distance of an integrin [nm]	30	(4)
**Sup**			
c	cytoskeleton		
e	extracellular matrix		
n	nucleus		
i	$i-th$ node		
t	transduce layer		
0	Previous time or initial state		
1	Present time		
**Sub**			
E	Elastic		
L	Lamellipodium		
FA	Focal adhesion		
SF	Stress fiber		
T	Transduce layer		
c	cytoskeleton		
e	extracellular matrix		
n	nucleus		
t	transduce layer		

|
